# Subcellular domain-dependent molecular hierarchy of SFK and FAK in mechanotransduction and cytokine signaling

**DOI:** 10.1038/s41598-017-09495-5

**Published:** 2017-08-22

**Authors:** Qiaoqiao Wan, ThucNhi TruongVo, Hannah E. Steele, Altug Ozcelikkale, Bumsoo Han, Yingxiao Wang, Junghwan Oh, Hiroki Yokota, Sungsoo Na

**Affiliations:** 1 Department of Biomedical Engineering, Indiana University-Purdue University Indianapolis, Indianapolis, Indiana, 46202 USA; 2 School of Biomedical Engineering, Purdue University, West Lafayette, Indiana, 47907 USA; 3 School of Mechanical Engineering, Purdue University, West Lafayette, Indiana, 47907 USA; 4 Department of Bioengineering, University of California San Diego, La Jolla, California, 92093 USA; 5 Department of Biomedical Engineering, Pukyong National University, Busan, 48513 Republic of Korea; 6 Department of Cell Biology, Present Address: Harvard Medical School, Boston, Massachusetts, 02115 USA

## Abstract

Focal adhesion kinase (FAK) and Src family kinases (SFK) are known to play critical roles in mechanotransduction and other crucial cell functions. Recent reports indicate that they reside in different microdomains of the plasma membrane. However, little is known about their subcellular domain-dependent roles and responses to extracellular stimuli. Here, we employed fluorescence resonance energy transfer (FRET)-based biosensors in conjunction with collagen-coupled agarose gels to detect subcellular activities of SFK and FAK in three-dimensional (3D) settings. We observed that SFK and FAK in the lipid rafts and nonrafts are differently regulated by fluid flow and pro-inflammatory cytokines. Inhibition of FAK in the lipid rafts blocked SFK response to fluid flow, while inhibition of SFK in the non-rafts blocked FAK activation by the cytokines. *Ex-vivo* FRET imaging of mouse cartilage explants showed that intermediate level of interstitial fluid flow selectively decreased cytokine-induced SFK/FAK activation. These findings suggest that SFK and FAK exert distinctive molecular hierarchy depending on their subcellular location and extracellular stimuli.

## Introduction

Mechanical stimuli are thought to be sensed by a cell via cell surface receptors such as integrins^1, 2^. When activated by mechanical loads, integrins undergo conformational changes^3, 4^ and increase their affinity to extracellular matrix (ECM) proteins as well as various intracellular focal adhesion proteins^5^. This integrin activation by mechanical stimulation is known to correlate with tyrosine phosphorylation of FAK and SFK^6^. They are considered as the main mechanotransduction signaling proteins at the cell-ECM adhesion sites and their activities influence various structural and signaling changes within the cell, including cytoskeletal organization, migration, proliferation, differentiation, and survival^7^. Accumulating evidence has shown that integrin-mediated signaling activities through SFK and FAK can regulate cell functions and pathology either cooperatively or independently. SFK and FAK form complexes, and lead to the activation of extracellular signal-regulated kinase (ERK) through the mitogen-activated protein kinase (MAPK) signaling pathway^8^. ERK activation in chondrocytes by fluid flow^9^ or compression^10^ has been reported to be associated with the regulation of both ECM gene expression and matrix metalloproteinase (MMP) activities. ERK activation by catabolic factors also induces cartilage degradation and inhibition of ERK reduces MMP activities^11^. In addition to the linkage of SFK and FAK to the regulation of ECM gene expression and MMP activities, they directly influence the cartilage pathology. It has been shown that FAK is up-regulated in both osteoarthritis and rheumatoid arthritis tissues^12^. FAK inhibition by siRNA transfection can decrease chondrocyte proliferation^13^. SFK inhibition has also been reported to reduce chondrocyte proliferation and promote chondrogenic gene expression, thus maintaining the chondrocyte phenotype^14^. Another study using rats with collagen-induced arthritis has shown that inhibiting SFK can reduce cartilage degradation^15^.

Because integrins are cell surface receptors, and FAK and SFK are closely associated with them, the plasma membrane is considered to be the primary activation site for FAK and SFK^16^. For example, in response to flow-induced shear stress, FAK is activated at focal adhesions^17^. Direct activation of integrin β1 alone is shown to be sufficient to activate FAK^18^. Localized mechanical force using a bead coated with fibronectin, which is known to bind to integrins, induces SFK activation at the plasma membrane^19^. However, recent evidence suggests that FAK and SFK can be differently regulated depending on their location within the membrane domains such as lipid and non-lipid rafts, and that the molecular relationship between these two proteins and their roles in the signaling pathways are distinct depending on the membrane microdomains^20, 21^. For example, SFK in the lipid rafts regulates the phosphoinositide 3-kinase (PI3K)/Akt signaling, whereas SFK in the non-lipid rafts regulates MAPK/ERK signaling^22^. The response of FAK in the lipid rafts to platelet-derived growth factor (PDGF) is much stronger and faster than that of FAK in the non-lipid raft regions^23^. During cell adhesion and spreading, FAK in the lipid rafts triggers activation of PI3K/Akt and controls early contact signaling, while FAK in the non-rafts subsequently triggers MAPK/ERK signaling and contribute to adhesion reinforcement^24^. Therefore, the mechanism of the domain-specific regulation of SFK and FAK by external stimuli, including mechanical force and growth factors, as well as their relationship, seems very complex. There is a need to understand this mechanism in various physiological and pathological conditions.

In addition to the responsiveness of FAK and SFK to mechanical force and growth factors, they are known to respond to pro-inflammatory cytokines such as tumor necrosis factor α (TNFα) and interleukin 1β (IL1β)^25^. Due to the involvement of FAK and SFK in inflammatory signaling, they are important in cartilage pathology. For example, phosphorylation of SFK and FAK elevates the gene transcription of MMPs^26, 27^. They also contribute to osteoarthritis progression by significantly elevating the expression of matrix degrading enzymes while inhibiting the gene expression of proteoglycan and type II collagen^28–30^. Despite the importance of FAK and SFK in inflammatory signaling, the detailed mechanism for the interaction between FAK and SFK activities, mechanical stimuli, and inflammatory cytokines in the different membrane domains (i.e., lipid rafts and non-rafts) is not known.

Current understanding of the cell behavior and signaling has been derived primarily from studying cells cultured on two-dimensional (2D) surfaces. However, it has been recently recognized that there are considerable differences in various cell functions between 2D and 3D extracellular environments, such as cell shape, differentiation, adhesion, migration, and force sensing^31, 32^. For example, when chondrocytes are isolated from articular cartilage and kept in planar 2D culture, they become flat and lose their cartilage phenotype^33^. When these “dedifferentiated” cells are cultured in 3D matrices, they become spherical, similar to their cell shape *in vivo*, and restore the “differentiated” cartilage phenotype^34^. Similarly, focal adhesion proteins, which play critical roles in mechanotransduction signaling and cytoskeletal organization, are highly expressed in 2D cultures, but are much less apparent *in vivo* and in 3D cultures^31, 35^. Therefore, a 3D culture model may more closely capture the physiological behavior of cells.

Here, we employed FRET-based biosensors to monitor SFK and FAK activities with high spatiotemporal resolution with different subcellular domains: the lipid rafts-targeting (Lyn-FAK and Lyn-SFK); and the nonrafts-targeting (KRas-FAK and KRas-SFK) biosensors. C28/I2 chondrocytes transfected with one of the biosensors were mixed with type II collagen-coupled agarose gel to produce 3D chondrocytes-gel constructs that allow integrin activation. During imaging, fluid flow or cytokines was applied to cells in 3D gel constructs. To examine the interactions between SFK and FAK, we used pharmacological drugs to specifically inhibit SFK or FAK activities. The role of Pyk2 in SFK/FAK signaling in response to mechanical or inflammatory cytokine stimulations was examined by silencing the Pyk2 activity using siRNA. A 3D cartilage explant system in conjunction with 3D FRET imaging was developed to further examine the effect of moderate loading on inflammatory cytokine-activated SFK/FAK signaling.

## Results

### Collagen-coupled agarose gels enhance mechanotransduction in 3D

**Figure 1:**
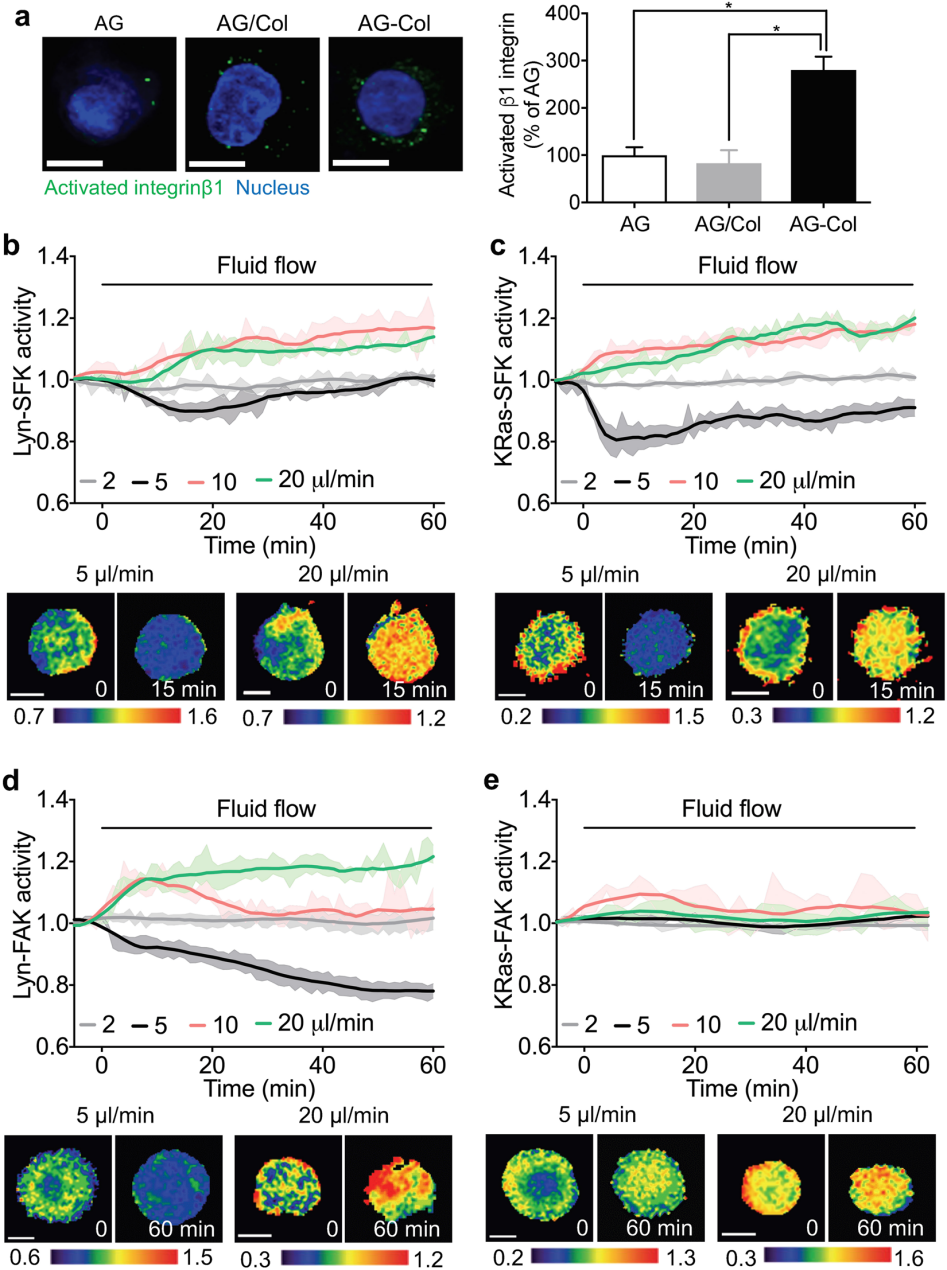
Subcellular domain-dependent activities of the SFK and FAK biosensors under interstitial fluid flow. (**a**) The representative immunostaining images of the activated β1 integrins (green) and nuclei (blue) in AG, AG/Col, and AG-Col. Scale bars, 10 μm. The bar graphs represent the GFP intensity of the activated β1 integrins averaged over the whole cells in AG (*n* = 11), AG/Col (*n* = 10), and AG-Col (*n* = 10). They were normalized against the averaged GFP intensity obtained in the AG. *p < 0.0001. (**b**–**e**) Cells were trasnsfected with one of the FRET biosensors, mixed with AG-Col, transferred to the flow chamber, and were subjected to interstitial fluid flow during imaging. Time courses represent normalized FRET ratios of the biosensors under flow. Representative FRET ratio images were scaled according to the corresponding color bar. Scale bars, 10 μm. (**b**) Lyn-SFK activities under flow (*n* = 9, 11, 9, 7 in 2, 5, 10, 20 μl/min). (**c**) KRas-SFK activities under flow (*n* = 7, 10, 9, 12 in 2, 5, 10, 20 μl/min). (**d**) Lyn-FAK activities under flow (*n* = 10, 7, 13, 9 in 2, 5, 10, 20 μl/min). (**e**) KRas-FAK activities under flow (*n* = 10, 11, 7, 9 in 2, 5, 10, 20 μl/min).

Here, we used agarose gels to mimic physiologically relevant 3D cellular microenvironment. Because integrin activation is required for the initiation of load-induced FAK/SFK signaling^36^, we set out to determine whether gels used in this study enable integrin activation. Three types of gels were tested: agarose gel (AG), type II collagen-added agarose gel (AG/Col), and type II collagen-crosslinked agarose gel (AG-Col). Immunostaining was employed to measure the β_1_ integrin activation levels of C28/I2 cells grown in agarose gels with three different modifications (Fig. 1a). The average green fluorescent protein (GFP) intensity over the whole cell was quantified and normalized to that in the AG. The collagen conjugation in the AG-Col significantly elevated the integrin activation level (180.3% increase) compared to the AG, while AG/Col did not induce integrin activation (Fig. 1a). The total (active + inactive) integrin levels in the three gel types were not significantly different (Supplementary Fig. S1a,b). These results indicate that the AG-Col promotes integrin activation, and thus is an excellent 3D extracellular matrix model for a mechanotransduction study. We next examined the flow characteristics of the AG-Col. The interstitial fluid flow in knee cartilage is reported to be 6–30 μm/min^37^ and 1.5 body weight generates interstitial fluid flow at 12 μm/min in human cartilage^38^. To evaluate the flow speed experienced by chondrocytes within 3D gels under various fluid flow rates, the gel was perfused with Alexa Fluor 594-conjugated bovine serum albumin (BSA). The fluorescence intensity change in the region of interest over time was monitored under flow to determine the flow speed in the gels (Supplementary Fig. S2a). We observed that application of 2–20 μl/min of fluid flow creates the flow speed at 4.75–29.4 μm/min (Supplementary Fig. S2b), similar to the reported range in knee cartilage. To estimate shear stress experienced by the cells in the 3D gels, we measured the Darcy permeability (Supplementary Fig. S2c). Based on the permeability values, the shear stress applied on cells through pores within the AG-Col under 2–20 μl/min flow was estimated to be ~0.16–1.6 Pa, similar to the frequently used range in chondrocyte studies *in vitro* ^25, 39^. The permeability values between AG/Col and AG-Col were not significantly different, indicating that collagen crosslinking did not significantly affect permeability.

To further evaluate the role of the collagen crosslinking in the gels in mechanosensitivity of SFK and FAK, we transfected cells with lipid raft-targeting FRET biosensors that are known to respond to mechanical stimuli^19, 20^. Application of 20 μl/min fluid flow (equivalent to 1.6 Pa shear stress) induced activation of both Lyn-SFK and Lyn-FAK of a cell grown in the AG-Col, while it did not alter the signaling activities in the AG and AG/Col (Supplementary Fig. S3). Moreover, the basal activities of Lyn-SFK and Lyn-FAK in the AG-Col are significantly higher than those in the AG and AG/Col. Thus, we decided to use the AG-Col for subsequent experiments in this study.

Because cells were spherical within the 3D gels, we tested whether FRET measurements of subcellular signaling activities are different depending on the viewing angle. Z-stack images of Lyn-SFK activities in the x-y plane were captured to create orthogonal view images in the x-z and y-z planes. The results revealed that the spatial distribution and level of the signaling activity were similar in all three different (x-y, x-z, and y-z) projections (Supplementary Fig. S4).

### Interstitial fluid flow regulates SFK and FAK in a flow magnitude-dependent manner

We have previously shown that SFK and FAK have distinct activation mechanisms depending on their subcellular locations^18–21, 25, 40^. To further explore the flow-driven activities of SFK and FAK at different subcellular locations (i.e., lipid rafts vs. non-rafts), we transfected cells with one of four biosensors: Lyn-SFK and Lyn-FAK that target lipid rafts, and KRas-SFK and KRas-FAK that target non-rafts. The transfected cells were embedded in AG-Col. We observed the FRET activities in response to different interstitial fluid flow rates (Fig. 1b–e). Note that the interstitial fluid flow was applied to the cells through pores within AG-Col. 2 μl/min fluid flow did not detectably alter Lyn-SFK activity (Fig. 1b). Notably, the intermediate 5 μl/min fluid flow reduced the Lyn-SFK activity (10.7% decrease at 14 min) and this inhibitory effect was reversed to the basal level at 60 min. Both 10 μl/min and 20 μl/min fluid flow significantly elevated Lyn-SFK activities. KRas-SFK activities were also downregulated by 5 μl/min (20.7% decrease at 6 min), while upregulated by 10 μl/min and 20 μl/min (Fig. 1c). Lyn-FAK activities were similarly regulated by different magnitudes of fluid flow, but its response to 5 μl/min was sustained during flow application (Fig. 1d). However, KRas-FAK activities were not detectably altered by interstitial fluid flow (Fig. 1e).

**Figure 2:**
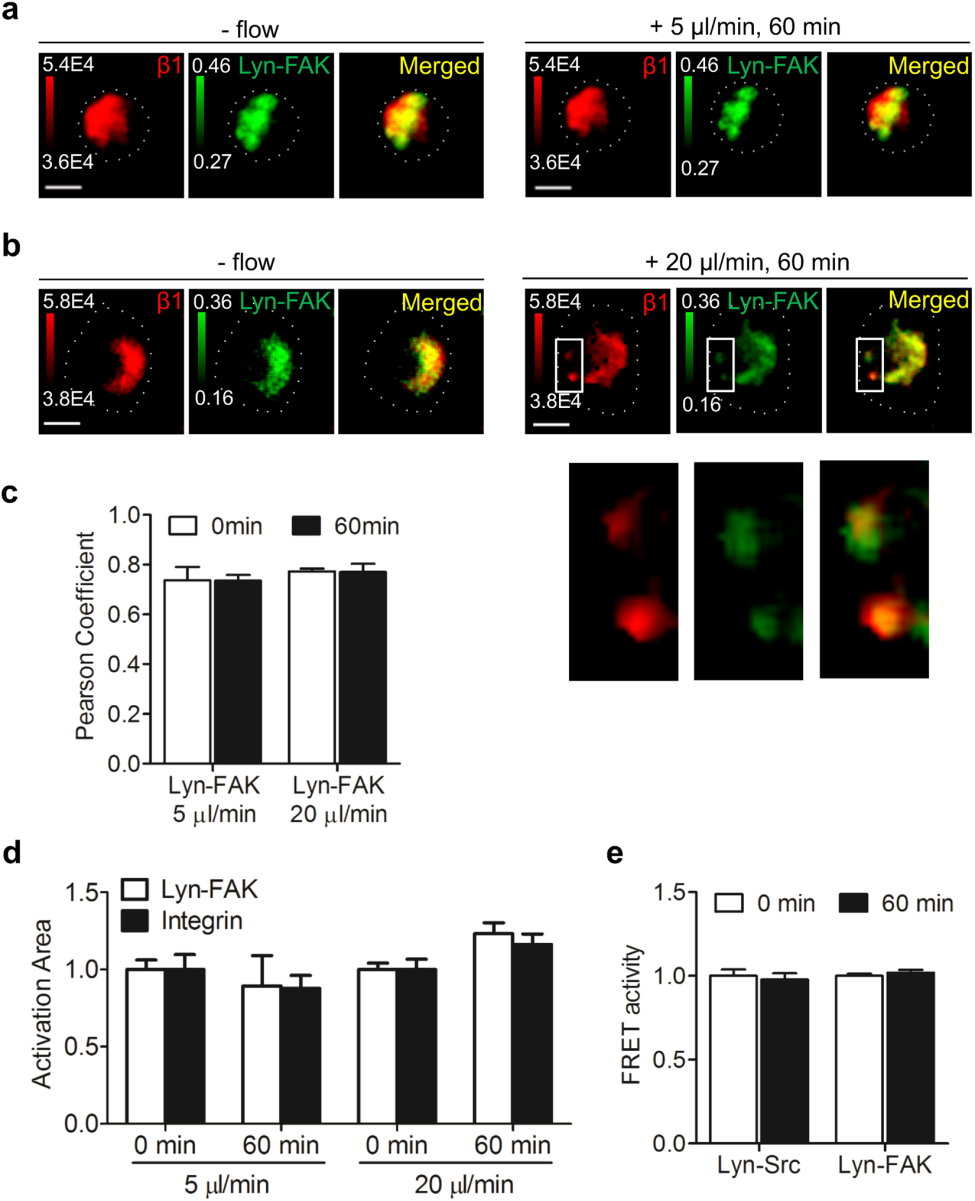
Integrin clustering and Lyn-FAK activity under flow. (**a**,**b**) The representative images of integrin β1 and Lyn-FAK activation before and after flow at 5 μl/min (**a**) and 20 μl/min (**b**). The integrin β1 images (*left*), originally 16 bit grayscale, were scaled according to the red colorbar with the thresholded range (see the values in the images). The Lyn-FAK activity images (*middle*) were produced in terms of the FRET ratio, and scaled according to the green colorbar. Merged images (*right*) indicate colocalization of integrin β1 and Lyn-FAK activation. Scale bars, 10 μm. (**c**) Pearson correlation coefficients between integrin β1 localization and Lyn-FAK activation based on the images obtained in (**a,b**). *n = *10. (**d**) Changes in integrin β1 and Lyn-FAK before (0 min) and after (60 min) flow application in terms of area normalized to 0 min. The area was quantified based on the images in (**a,b**). *n = *10. (**e**) Lyn-SFK (*n* = 9) and Lyn-FAK (*n* = 10) activity of the cell pretreated with a function-blocking antibody against β1 integrins before (0 min) and after (60 min) 20 μl/min fluid flow.

Because integrin clustering and FAK activation in the lipid rafts are closely linked^41, 42^ and FAK binds to the cytoplasmic domain of β integrin subunits^43^, we postulated that the flow magnitude-dependent FAK activities would be correlated to changes in integrin clustering. To test this hypothesis, we double transfected cells with Lyn-FAK and mCherry-β1 integrin. Using confocal microscopy, images of FAK activities and β1 integrins were obtained before and after 1 h application of fluid flow from the same cell. We observed that the FAK activation sites colocalized with β1 integrin sites regardless of interstitial fluid flow application (Fig. 2a–c). This result indicates that integrin β1 might be involved in basal Lyn-FAK activity as well as flow-induced Lyn-FAK activity. The representative images in Fig. 2a show that 5 μl/min fluid flow tended to decrease the size of Lyn-FAK activation and integrin β1 clustering sites. In contrast, 20 μl/min fluid flow increased the size of activation sites of Lyn-FAK and integrin β1 localization, and the newly created Lyn-FAK activation sites colocalized with new integrin β1 clustering sites (Fig. 2b). Quantitative analysis using the Pearson’s correlation coefficient^44^ also shows the strong colocalization of Lyn-FAK and integrin β1 (Fig. 2c). While low (5 μl/min) and high (20 μl/min) interstitial fluid flows tended to decrease and increase the size of colocalized sites, respectively, the activation area did not significantly change under different flow conditions (Fig. 2d). We also tested whether flow-induced SFK/FAK signaling is dependent on integrins. We first transfected cells with a Lyn-SFK or Lyn-FAK biosensor. Before imaging, cells were preincubated with a function-blocking antibody against β1 integrins. During imaging, a 20 μl/min flowrate was applied to the cells. The flow-induced Lyn-SFK and Lyn-FAK activities were not altered by flow, suggesting that β1 integrins are required for flow-induced activation of SFK and FAK (Fig. 2e).

### Activation of SFK and FAK by TNFα and IL1β at different subcellular microdomains

**Figure 3:**
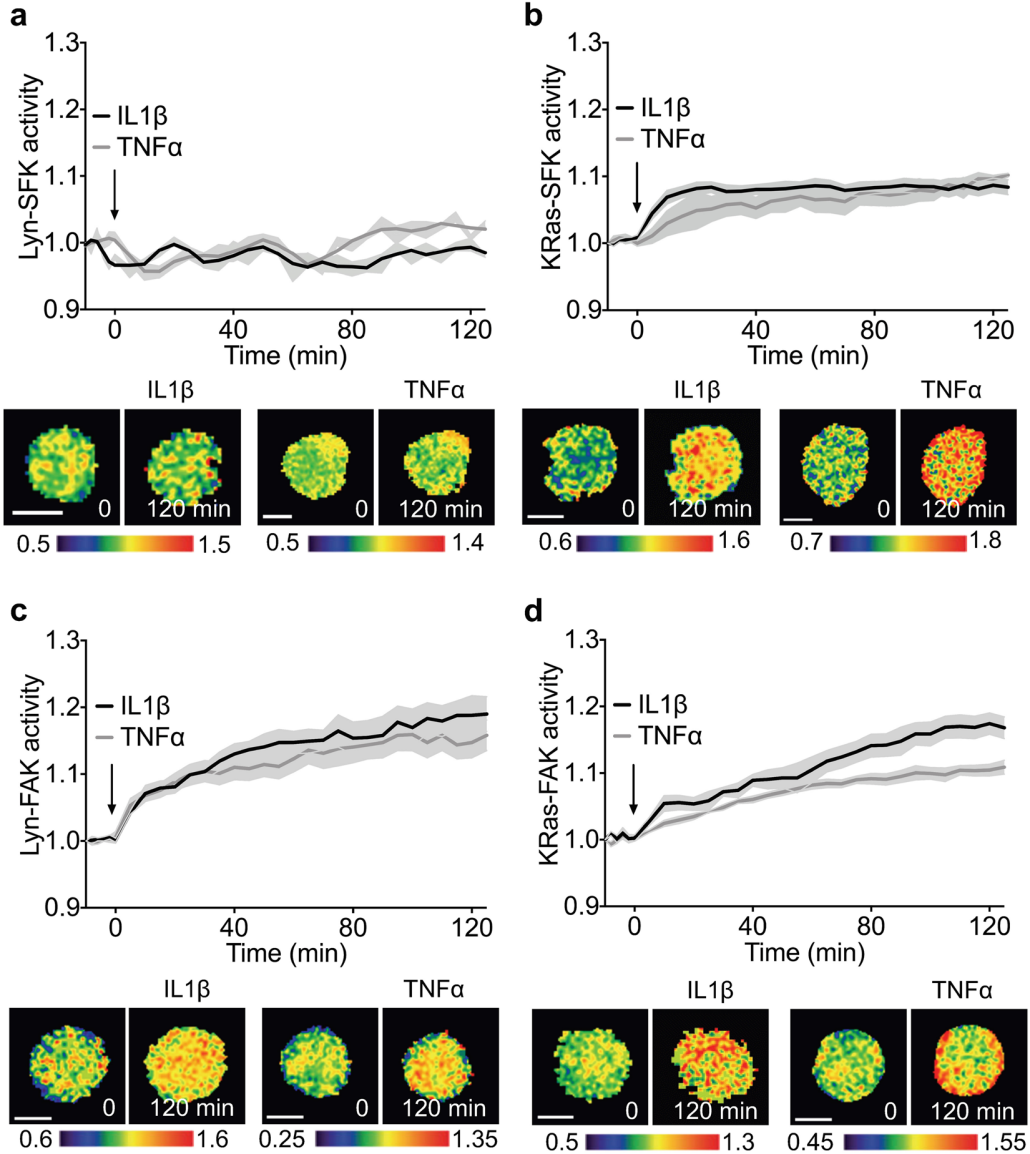
Subcellular domain-dependent activities of SFK and FAK under IL1β and TNFα. Cells transfected with one of the FRET biosensors were imaged under treatment of either 1 ng/ml IL1β or 10 ng/ml TNFα. (**a**) Lyn-SFK activities (*n* = 6 in IL1β, *n* = 6 in TNFα). (**b**) KRas-SFK activities (*n* = 7 in IL1β, *n* = 6 in TNFα). (**c**) Lyn-FAK activities (*n* = 6 in IL1β, *n* = 6 in TNFα). (**d**) KRas-FAK activities (*n* = 6 in IL1β, *n* = 6 in TNFα). Time courses represent normalized FRET ratios of the biosensors under the cytokine. The representative FRET ratio images were scaled according to the color bar. Scale bars, 10 μm.

To examine SFK and FAK activities in response to pro-inflammatory cytokines, C28/I2 cells transfected with one of the FRET biosensors were imaged for 2 h under the treatment of IL1β (1 ng/ml) or TNFα (10 ng/ml). We observed that Lyn-SFK activities were not significantly altered under cytokine treatment (Fig. 3a). KRas-SFK activities, however, were significantly increased under the cytokines (Fig. 3b). These results are consistent with our previous report on differential Lyn-SFK and KRas-SFK activities in response to growth factors (epidermal growth factor and platelet-derived growth factor)^21^. KRas-SFK was significantly upregulated and reached the peak value (8.4%) at 25 min under IL1β, while it reached the maximal (10.2%) at 120 min under TNFα (Fig. 3b). Both Lyn-FAK and KRas-FAK were significantly activated by the cytokines within 10–25 minutes and reached the peak values at 120 min (Lyn-FAK + TNFα: 15.8%; Lyn-FAK + IL1β: 19.0%; KRas-FAK + TNFα: 10.9%; KRas-FAK + IL1β: 16.8%) (Fig. 3c,d). These results suggest that SFK and FAK may have different activation mechanisms in response to pro-inflammatory cytokines and that SFK in the lipid rafts at the plasma membrane may not be involved in pro-inflammatory cytokine-induced signaling.

### SFK and FAK exert distinct molecular hierarchy in response to flow and cytokines

**Figure 4:**
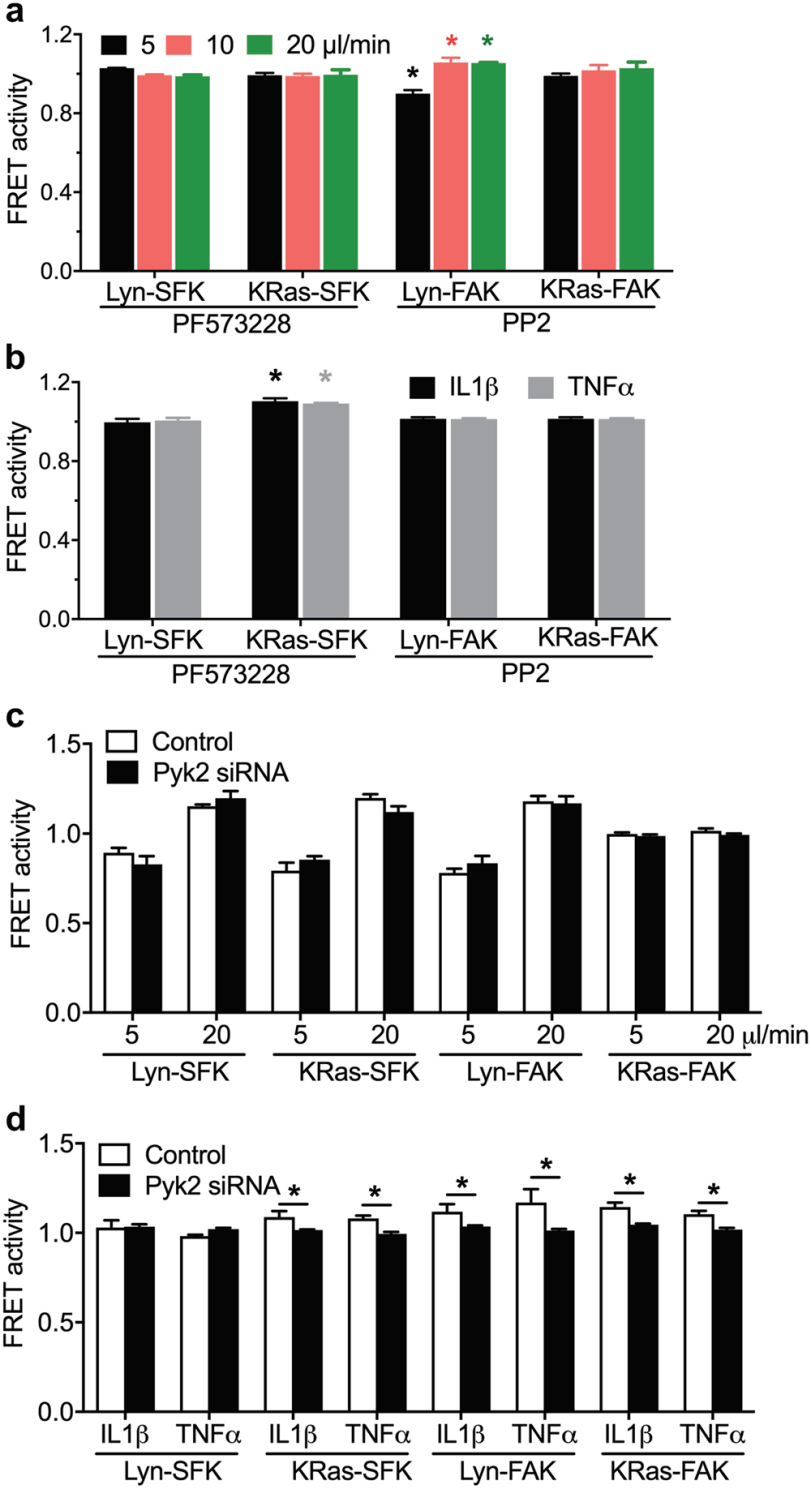
Distinct molecular hierarchy of SFK and FAK under flow and pro-inflammatory cytokines. (**a**) The flow-driven activities of SFK and FAK at different subcellular domains in cells pretreated with FAK and SFK inhibitors. Cells transfected with either a Lyn-SFK or KRas-SFK biosensor were pretreated with PF573228 (1 μM, 1 hour). Cells transfected with either a Lyn-FAK or KRas-FAK were pretreated with PP2 (10 μM, 1 hour). The bar graphs represent SFK and FAK activities at 60 min after the application of fluid flow (5, 10, or 20 μl/min). The activities were normalized to those at 0 min. *p < 0.05 compared to the corresponding FRET activities at 0 min. Lyn-SFK under PF573228, *n* = 6, 7, 7 in 5, 10, 20 μl/min; KRas-SFK under PF573228, *n* = 8, 8, 8 in 5, 10, 20 μl/min; Lyn-FAK under PP2, *n* = 8, 10, 10 in 5, 10, 20 μl/min; KRas-FAK under PP2, *n* = 8, 10, 10 in 5, 10, 20 μl/min. (**b**) The pro-inflammatory cytokine-induced activities of SFK and FAK at different subcellular domains in cells pretreated with FAK and SFK inhibitors. Cells transfected with either a Lyn-SFK or KRas-SFK biosensor were pretreated with PF573228. Cells transfected with either a Lyn-FAK or KRas-FAK biosensor were pretreated with PP2. The bar graphs represent SFK and FAK activities at 120 min after the addition of the cytokines (10 ng/ml TNFα or 1 ng/ml IL1β). The activities were normalized to those at 0 min. *p < 0.01 compared to the corresponding FRET activities at 0 min. Lyn-SFK under PF573228, *n* = 7, 7 in IL1β and TNFα; KRas-SFK under PF573228, *n* = 7, 10 in IL1β and TNFα; Lyn-FAK under PP2, *n* = 8, 11 in IL1β and TNFα; KRas-FAK under PP2, *n* = 10, 10 in IL1β and TNFα. (**c**) The flow-driven activities of SFK and FAK at different subcellular domains in cells pretreated with either non-specific control (NC) siRNA or Pyk2 siRNA. Cells were co-transfected with either NC or Pyk2 siRNA and one of FRET biosensors, and then subjected to 5 or 20 μl/min fluid flow for 1 hour. The bar graphs represent SFK and FAK activities at 60 min after flow application. The activities were normalized to those at 0 min. Lyn-SFK under 5 μl/min, *n* = 6, 6 in NC, Pyk2 siRNA; Lyn-SFK under 20 μl/min, *n* = 6, 6 in NC, Pyk2 siRNA; KRas-SFK under 5 μl/min, *n* = 10, 8 in NC, Pyk2 siRNA; KRas-SFK under 20 μl/min, *n* = 12, 8 in NC, Pyk2 siRNA; Lyn-FAK under 5 μl/min, *n* = 6, 8 in NC, Pyk2 siRNA; Lyn-FAK under 20 μl/min, *n* = 9, 10 in NC, Pyk2 siRNA; KRas-FAK under 5 μl/min, *n* = 6, 8 in NC, Pyk2 siRNA; KRas-FAK under 20 μl/min, *n* = 8, 10 in NC, Pyk2 siRNA. (**d**) The pro-inflammatory cytokine-induced activities of SFK and FAK at different subcellular domains in cells pretreated with either NC or Pyk2 siRNA. Cells were co-transfected with either NC or Pyk2 siRNA and one of FRET biosensors and incubated with the cytokines. The bar graphs represent SFK and FAK activities at 120 min after the addition of the cytokines (1 ng/ml IL1β or 10 ng/ml TNFα). The activities were normalized to those at 0 min. *p < 0.01 compared to the corresponding FRET activities at 0 min. Lyn-SFK under IL1β, *n* = 6, 6 in NC, Pyk2 siRNA; Lyn-SFK under TNFα, *n* = 6, 9 in NC, Pyk2 siRNA; KRas-SFK under IL1β, *n* = 7, 6 in NC, Pyk2 siRNA; KRas-SFK under TNFα, *n* = 6, 10 in NC, Pyk2 siRNA; Lyn-FAK under IL1β n, *n* = 6, 7 in NC, Pyk2 siRNA; Lyn-FAK under TNFα, *n* = 7, 11 in NC, Pyk2 siRNA; KRas-FAK under IL1β, *n* = 6, 7 in NC, Pyk2 siRNA; KRas-FAK under TNFα, *n* = 7, 12 in NC, Pyk2 siRNA.

We explored the interaction between SFK and FAK in response to fluid flow. First, cells transfected with one of the SFK biosensors were pretreated with PF573228. The activities of Lyn-SFK and KRas-SFK at 0 and 60 min were recorded under flow, and were normalized to those at 0 min. The results revealed that the fluid flow-induced Lyn-SFK and KRas-SFK activities were abolished by the pretreatment of PF573228 (Fig. 4a), suggesting that FAK is necessary for SFK mechanotransduction. Next, cells were transfected with one of the FAK biosensors and pretreated with PP2. In contrast to the SFK activity of the cells pretreated with a FAK inhibitor, PP2 treatment did not prevent the Lyn-FAK activities under flow and these flow magnitude-dependent activities showed a similar trend to the non-PP2-treated Lyn-FAK activities observed in Figs 1d and 4a). As expected, the KRas-FAK, pre-treated with PP2, was not significantly responsive to fluid flow, similar to the non-responsive KRas-FAK under flow (see Fig. 1e).

Although our results indicate that FAK inhibition abolished the activities of both Lyn-SFK and KRas-SFK under flow, the results could not rule out the possibility of the interaction of SFK and FAK between the different subcellular microdomains (i.e., lipid rafts and non-rafts) at the plasma membrane. To further explore this, we transfected cells with either a Lyn-FAK or a KRas-FAK biosensor and measured the FRET response to PF573228. Our results (Supplementary Fig. S5a) showed that Lyn-FAK was downregulated by PF573228, but KRas-FAK did not respond to the drug, suggesting that FAK activity is higher in the lipid raft than the non-raft regions. This result is consistent with our previous report^20^. Thus, we postulated that FAK at the lipid rafts (Lyn-FAK) would be responsible for the abolished KRas-SFK response to flow. To test this hypothesis, we transfected cells with a KRas-SFK biosensor and pretreated with methyl-β-cyclodextrin (MβCD) which extracts cholesterol and disrupts the lipid rafts of the plasma membrane^41^. The fluid flow-driven KRas-SFK activities were blocked by the treatment of MβCD (Supplementary Fig. S6). This result suggests that FAK in the lipid rafts is essential for both Lyn- and KRas-SFK activities in response to fluid flow.

We also examined the interaction between SFK and FAK under TNFα and IL1β. First, we tested the involvement of FAK in the cytokine-induced SFK. Cells transfected with one of the SFK biosensors were pre-incubated with PF573228. The activities of Lyn- and KRas-SFK at 0 and 120 min were recorded, and were normalized to those at 0 min. As shown in Fig. 4b, the Lyn-SFK pre-treated with PF573228 was not responsive to the cytokines, similar to the non-responsive Lyn-SFK under the cytokines (see Fig. 3a). Interestingly, the pretreatment of PF573228 did not block KRas-SFK activation by the cytokines (Fig. 4b, Supplementary Fig. S7a). Next, to test the involvement of SFK in FAK activation by the cytokines, cells were transfected with one of the FAK biosensors and were pretreated with PP2. The activation of Lyn-FAK and KRas-FAK by the cytokines, previously observed in Fig. 3, were completely blocked by the treatment of PP2 (Fig. 4b, Supplementary Fig. S7a). Because Lyn-SFK was shown to be unresponsive to the cytokines (see Fig. 3a), SFK in the non-rafts appears to be essential for pro-inflammatory cytokine-induced FAK activation. Taken together, these results suggest that SFK and FAK may have different molecular hierarchy in response to fluid flow and pro-inflammatory cytokines.

### Pyk2 is necessary for cytokine-induced SFK/FAK activation

Pyk2 (Proline-rich tyrosine kinase 2) is another tyrosine kinase whose sequence and structure are similar to those of FAK, and it binds to SFK via the SH2 domain of SFK^45^. Because of the similarity between Pyk2 and FAK, we first tested whether FAK is involved in Pyk2 activation. Western blotting revealed that incubation with PF573228 did not alter the phosphorylation level of Pyk2 (p-Pyk2; Supplementary Fig. S7b). In order to investigate the role of Pyk2 in the flow-driven SFK and FAK activities, cells were co-transfected with Pyk2 siRNA (Supplementary Fig. S7c) and one of the FRET biosensors. SFK or FAK activities were recorded at 0 and 60 min under flow application, and were normalized to those at 0 min (Fig. 4c, Supplementary Fig. S7d). No significant difference was observed between the control and Pyk2 siRNA groups under all flow conditions, suggesting that Pyk2 is not involved in the flow-driven activities of FAK and SFK. We also examined the role of Pyk2 in SFK and FAK activation by IL1β and TNFα (Fig. 4d). While silencing of Pyk2 by siRNA did not affect the basal level of FAK activity (Supplementary Fig. S8), it prevented the activation of KRas-SFK, Lyn-FAK, and KRas-FAK by the cytokines. Lyn-SFK was unresponsive to the cytokines with and without Pyk2 silencing, consistent with the results in Fig. 3a. Taken together, the results suggest that Pyk2 is involved in the cytokine-induced SFK/FAK signaling, but not in the flow-induced mechanotransduction of SFK and FAK.

### Intermediate fluid flow selectively suppresses cytokine-induced SFK/FAK activation in mouse cartilage explants

**Figure 5:**
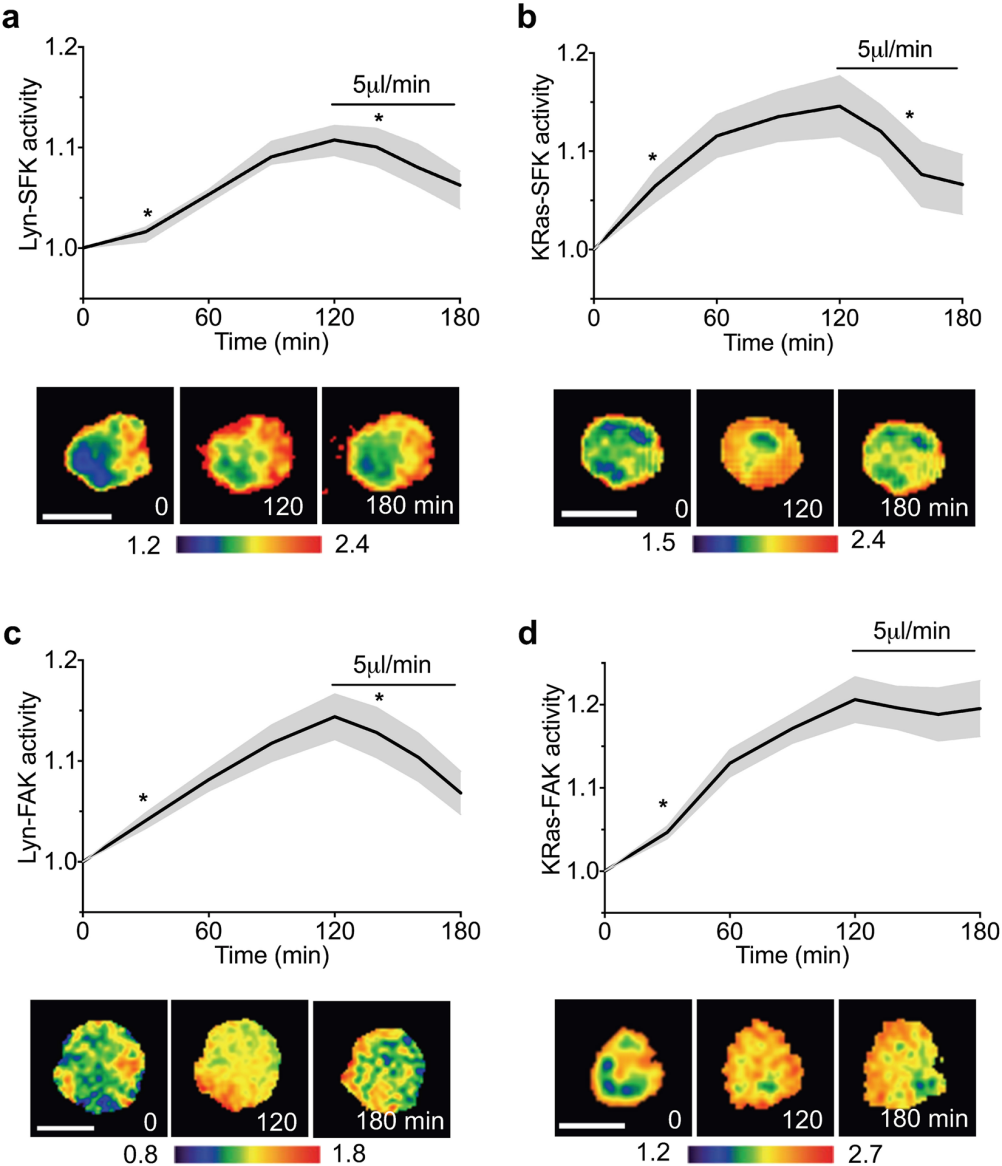
Intermediate level of fluid flow enables suppression of IL1β-stimulated activation of Lyn-SFK, KRas-SFK and Lyn-FAK, but not KRas-FAK. Chondrocytes in mouse cartilage explants were transfected with one of the FRET biosensors. During imaging, cells were treated with 1 ng/ml IL1β for 2 hours (0–120 min) and then subjected to 5 μl/min fluid flow for 1 hour (120–180 min). The time course activities were normalized to the activities at 0 min. In the time course, * between 0 and 120 min (+IL1β) indicates the time point after which the activity becomes significantly different from that at 0 min (-IL1β), and * between 120 and 180 (+IL1β, +flow) min indicates the time point after which the activity becomes significantly different from that at 120 min (+IL1β, -flow). Representative FRET ratio images were scaled according to the corresponding color bar. Scale bars, 10 μm. (**a**) Lyn-SFK activity (*n* = 10). (**b**) KRas-SFK activity (*n* = 13). (**c**) Lyn-FAK activity (*n* = 18). (**d**) KRas-FAK activity (*n* = 15).

Using 3D cell/AG-Col constructs, we observed that Lyn-SFK, KRas-SFK, and Lyn-FAK were downregulated by intermediate, 5 μl/min interstitial fluid flow. To further examine whether this flow is able to reduce cytokine-induced SFK/FAK activation in more physiologically relevant settings, we used cartilage explants obtained from mice and transfected chondrocytes in the explants with one of the FRET biosensors. During FRET imaging, explants were treated with IL1β for 2 hours and then were subsequently subjected to 5 μl/min fluid flow for 1 hour. Interestingly, Lyn-SFK in the explants was significantly activated by IL1β (Fig. 5a), which was different from the results obtained from the *in vitro* experiments using cell/AG-Col constructs (see Fig. 3a). This might be due to the different cell type or ECM contributions to the Lyn-SFK response in agarose gels and native explant tissues. KRas-SFK, Lyn-FAK, and KRas-FAK activities were significantly upregulated by IL1β (Fig. 5b–d), similar to the results obtained from cell/AG-Col constructs (see Fig. 3b–d). The cytokine-induced upregulation was subsequently reduced in Lyn-SFK, KRas-SFK and Lyn-FAK, but not in KRas-FAK (Fig. 5). The fluid flow-driven SFK/FAK activities shown in the *ex vivo* explants were consistent with the results in cell/AG-Col constructs (see Fig. 1). Taken together, these results suggest that moderate fluid flow may selectively suppress cytokine-induced inflammatory signaling in SFK and FAK depending on their subcellular locations in the plasma membrane.

## Discussion

In this study, we employed collagen-conjugated 3D agarose gels in conjunction with live cell imaging to visualize the real-time activities of SFK and FAK in C28/I2 human chondrocytes. An agarose gel has been frequently used as a 3D cell culture scaffold due to its high biocompatibility, high water content and the presence of porous structure^46, 47^. It has been shown to maintain the chondrocyte phenotype and improve ECM synthesis^48–51^. However, this gel lacks ECM proteins, such as collagen, which may induce cell matrix interaction and subsequent integrin activation and integrin-dependent signaling. While the protein-conjugated agarose gel has been used in several studies^52, 53^ and there have been efforts to modify agarose gels using synthetic peptides (e.g., RGD) to promote cellular mechanosensing^54, 55^, the effect of ECM proteins on integrin activation and subsequent signaling in agarose gels has not been fully demonstrated. Here we show that the integrin activation level in cells seeded in the AG-Col was significantly higher than that in the AG and AG/Col. The integrin activation levels in different gels correlated well with the basal FRET activities of Lyn-FAK and Lyn-SFK. In addition, the cells in AG-Col, pretreated with a function-blocking integrin β1 antibody, had similar basal activities of Lyn-Src and Lyn-FAK, compared to those in AG and AG/Col. These results further suggest that integrin β1 is necessary to maintain a certain level of basal activities of FAK and SFK, which may be required for their response to extracellular stimuli such as interstitial fluid flow. Since integrin clustering and activation are closely associated with lipid rafts in the plasma membrane^56–58^, we used the FRET-based Lyn-SFK and Lyn-FAK biosensors targeting lipid rafts to determine whether the gels used in this study enable integrin-mediated activation of SFK and FAK under flow. While the FAK biosensor used in this study is only specific to FAK^20^, the SFK biosensor is specific to Src and moderately to Fyn^19^. Because Fyn has been shown to play a primary role in mechanotransduction^59, 60^, it is possible that both Src and Fyn might be involved in this interstitial fluid flow-driven mechanotransduction. Our data, based on live cell imaging of SFK and FAK and immunostaining of integrin β1 (Fig. 1) as well as live cell imaging of Lyn-FAK and integrin β1 (Fig. 2), strongly indicate that collagen conjugated agarose gels promote integrin activation and mechanotransduction of FAK and SFK.

Recent studies suggest that SFK and FAK at different subcellular locations may be regulated by distinct mechanisms^20–22^. While they are known to localize to various compartments within the cell, our focus was evaluation of them in the plasma membrane because mechanotransduction and cytokine signaling initiate from the plasma membrane^2, 61, 62^. Here, we show that they respond differently depending on their subcellular locations of plasma membrane and the type of extracellular stimuli. We observed that, except for KRas-FAK, the activities of Lyn-SFK, KRas-SFK, and Lyn-FAK are flow magnitude-dependent: moderate (5 μl/min) fluid flow decreased the activities, while high (20 μl/min) fluid flow increased the activities. This result is consistent with previous reports on loading magnitude-dependent activities of small GTPase RhoA^39^ and Src^25^ in 2D culture, as well as MMP13^63^ in animal models. While the articular cartilage is subjected to complex mechanical stimuli, including compression, fluid flow-induced shear stress, and hydrostatic pressure, etc., fluid flow is shown to play a crucial role in the cartilage maintenance and osteoarthritis progression^64^. For example, intermediate level of shear stress decreased small GTPase Rac1 activity in chondrocytes, while high shear stress increased it, and the reduction of Rac1 downregulated MMP 13 activity^63^. In this study, we observed that intermediate level of shear stress decreased FAK and SFK activities, which have been reported to be beneficial in maintaining cartilage integrity^14, 65^.

**Figure 6:**
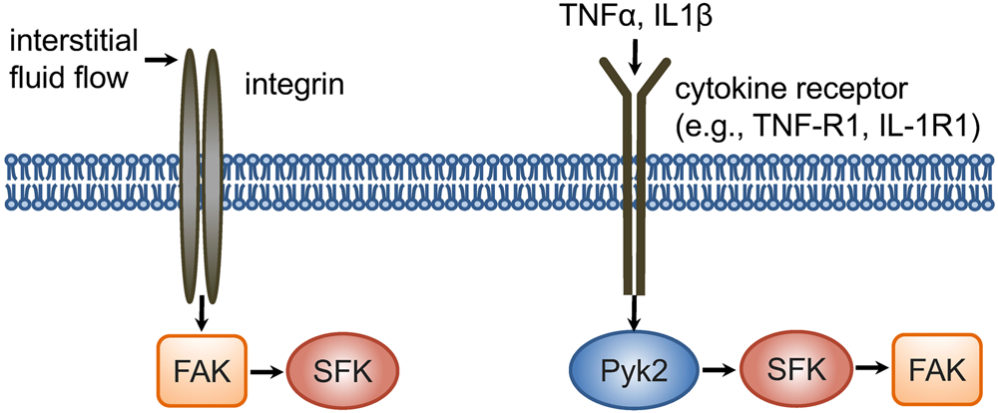
A proposed model of distinct molecular hierarchy of SFK and FAK in mechanotransduction and cytokine signaling. Interstitial fluid flow regulates FAK in the lipid rafts, which is mediated by integrin β1, and subsequently regulates SFK. In contrast, TNFα and IL1β activate SFK in the lipid rafts, which is mediated by Pyk2, and subsequently activate FAK. Through the integrin β1-FAK-SFK signaling axis, intermediate level of fluid flow enables suppression of pro-inflammatory cytokine-induced SFK/FAK signaling.

What is the mechanism responsible for the distinct activities of FAK and SFK at different subcellular domains? Earlier work demonstrated that lipid rafts are involved in integrin activation^41^. Our results also show that β1 integrins are required for flow-induced activation of SFK and FAK in lipid rafts, and the activation sites of FAK in lipid rafts are highly colocalized with those of β1 integrin clustering. Importantly, we observed that FAK is necessary for load-induced SFK activation in the lipid rafts as well as non-lipid rafts, while SFK is not required for fluid flow-driven FAK activities. It appears that FAK in the lipid rafts that closely interacts with integrins is essential to flow-induced SFK signaling (Fig. 6). This proposed mechanism was further confirmed by pretreating cells with MβCD that disrupts lipid rafts, which consequently blocked KRas-SFK activity in the non-rafts under fluid flow. In contrast to the effect of fluid flow, our study suggests that upon stimulation by TNFα and IL1β, phosphorylated Pyk2 creates a binding site for SH2 domain of SFK, resulting in SFK activation, and subsequent activation of FAK (Fig. 6). Both TNF receptors (TNF-R1) and IL1 receptors (IL-1R1) exist in the lipid rafts of the plasma membrane, and the signaling by TNFα and IL1β is reported to initiate from the lipid rafts^61, 62^. Therefore, the distinct response of FAK and SFK between mechanotransduction and cytokine signaling, presented in this study, may be due to the different extracellular stimuli (i.e., mechanical stimuli and cytokines) because this distinct response initiates from the same membrane compartment (i.e., lipid rafts). It is not clear whether Lyn-FAK is mediated by SFK in the non-rafts because both Lyn-SFK and KRas-SFK were downregulated by PP2 (see Supplementary Fig. S5b). Inhibition of SFK and FAK at specific microdomains of the plasma membrane will provide more details on the mechanisms and interactions of SFK and FAK.

In addition to the 3D agarose-based constructs, we used cartilage explants obtained from mice for live cell imaging of SFK/FAK activities. The results revealed that KRas-SFK, Lyn-FAK and KRas-FAK in the explants have similar responses to those obtained from the AG-Col constructs. However, we observed a discrepancy between the 3D *in vitro* and *ex vivo* data for the Lyn-SFK response to IL1β. Lyn-SFK was not significantly responsive to the cytokine *in vitro*, while it was substantially activated in the *ex vivo* model. Two possible factors might contribute to this discrepancy. First, the chondrocytes used in the AG-Col were C28I2 human cell line, but the explants employed in this study were obtained from mice. Cell types in different species might result in these different responses to cytokines. Second, different microenvironments might lead to the distinct response. The AG-Col includes only one type of collagen (type II), which is a major constituent of cartilage. Mouse cartilage explants include other ECM proteins that are known to play important roles in mechanotransduction, such as fibronectin^66^ and collagen VI^67^. These differential ECM constituents might lead to different cell-ECM interactions and subsequent integrin activation and SFK/FAK signaling.

In summary, our findings on the activities of FAK and SFK at the different microdomains of the plasma membrane suggest that their responses to mechanical forces and cytokines are governed by the distinct mechanisms. The results using mouse cartilage explants demonstrate that moderate mechanical stimuli are considered as a potential therapeutic regimen for prevention of OA progression due to excessive cytokine expression. By downregulating inflammatory cytokine-induced activation of SFK and FAK, moderate fluid flow and Pyk2 inhibition appear to promote cartilage health. Further studies on the regulatory pathways of SFK/FAK signaling and their linkage to gene expression profiles responsible for tissue homeostasis and maintenance will have important implications in skeletal health and diseases.

## Methods

### Biosensors and plasmids

FRET-based biosensors used for monitoring subcellular SFK and FAK activities were previously developed and described^19–21^. The SFK biosensor consists of a cyan fluorescent protein (CFP), the SH2 domain from c-SFK, a truncated SFK substrate peptide and a yellow fluorescent protein (YFP)^19^. The FAK biosensor consists of a CFP, the SH2 domain, a FAK substrate peptide and a YFP^20^. The lipid raft-targeting biosensors (Lyn-SFK and Lyn-FAK) were constructed by attaching a raft-targeting motif, derived from Lyn kinase, to the N-terminus of the biosensors^20, 21^. The non-lipid raft-targeting biosensors (KRas-SFK and KRas-FAK) were produced by attaching a non-raft targeting motif, derived from KRas, to the C-terminus of the biosensors^20, 21^. The specificity of the biosensors has been well characterized to represent corresponding endogenous protein activity^19–21, 25, 40^. The FAK biosensor is only specific to FAK, but not Pyk2^20^. The SFK biosensor is mostly specific to Src and moderately to Fyn but not Yes^19^. mCherry-integrin-beta1 was a gift from Michael Davison (Addgene).

### Reagents

Two types of pro-inflammatory cytokines, TNFα-human and TNFα-mouse (10 ng/ml; Sigma) as well as IL1β-human and IL1β-mouse (1 ng/ml; Sigma), were used. A function-blocking integrin β1 antibody (clone P5D2; 10 µg/ml; Santa Cruz Biotechnology) was used to block integrin activities. MβCD (10 mM; Sigma) was used to extract cholesterol from the lipid rafts of the plasma membrane. PP2 (10 µM; Sigma) was used to block SFK activities, and PF573228 (1 µM; Sigma) was used to inhibit FAK activities. Pyk2 siRNA and non-specific control (NC) siRNA (Santa Cruz Biotechnology) were used to investigate the role of Pyk2 in SFK and FAK activities.

### Agarose-based gels

The collagen-coupled agarose gels (AG-Col) were prepared as previously described^52^. Briefly, the type II collagen (Sigma) was suspended at 1.2 mg/ml in acetic acid (0.1 M), and was mixed with a 10-fold molar excess of the N-Sulfosuccinimidyl-6-(4′-azido-2′-nitrophenylamino) hexanoate (Sulfo-SANPAH; Thermo Fisher Scientific) in PBS at room temperature in the dark for 4 h. The 4% (w/v) agarose solution was prepared by dissolving low melting temperature agarose (Lonza) in sterile PBS. Three parts 4% agarose solution was then mixed with one part collagen/Sulfo-SANPAH solution to yield the mixture containing 3% agarose and 0.3 mg/ml collagen. The mixture was exposed under UV light for 20 min to allow the photocrosslinking reaction and conjugation of the collagen to the agarose. After conjugation, the agarose mixture was cooled down and washed with sterile PBS for 4 days to remove the unbound collagen and unreacted Sulfo-SANPAH. For control gels, AG/Col was prepared as stated previously without the addition of the Sulfo-SANPAH, and AG was prepared without the addition of the collagen and Sulfo-SANPAH.

### Cell culture, transfection, and Western blotting

The human chondrocyte cell line C28/I2 was used^68^. Cells were cultured in Dulbecco’s modified Eagle’s medium (DMEM; Lonza) containing 10% FBS (Hyclone) and 1% penicillin/streptomycin (Lonza), and maintained at 37 °C and 5% CO_2_ in a humidified incubator. Neon transfection system (Invitrogen) was used to transfect the biosensors into the cells. After transfection, two parts 3% agarose gel solution was mixed with one part 3 × DMEM containing transfected cells to produce the cell/gel construct with 2% agarose and 1 × DMEM. The mixture was injected into the μ-slide flow chamber (Ibidi) and cooled at room temperature for 30 min to allow gelation. The cell/gel construct was incubated in DMEM containing 0.5% FBS for 24 h before imaging 7 experiments. For Western blot analysis, cells were lysed in a radioimmunoprecipitation assay (RIPA) buffer. Isolated proteins were fractionated using 10% SDS gels and electro-transferred to Immobilon-P membranes. The membrane was incubated with primary antibodies, followed by incubation with secondary antibodies conjugated with horseradish peroxidase (Cell Signaling). The primary antibodies used were Pyk2 (Cell Signaling), phospho-Pyk2 (Tyr402) (Cell Signaling), FAK (Cell Signaling), phospho-FAK (Tyr397) (Cell Signaling), and β-actin (Sigma). Signal intensities were quantified by a luminescent image analyzer (Fujifilm).

### Interstitial fluid flow application

During imaging, interstitial fluid flow was applied to the cell/gel construct within the flow chamber with phenol red-free and serum-free DMEM. Here, fluid flow was applied through pores within the gel in which cells were embedded. An environmental chamber (Ibidi) was used to maintain temperature (37 °C) and CO_2_ level (5%). A programmable syringe pump (Harvard Apparatus) was used to apply interstitial fluid flow to the cells by controlling the flow rate. A pulsatile (0.2 Hz) flow at four different flow rates (2, 5, 10, and 20 μl/min) was used.

### Characterization of the cell/gel constructs

The flow speed within the cell/gel construct was obtained by monitoring the intensity of fluorescent molecules traveling through the agarose gels during flow application. Alexa Fluor 594-conjugated bovine serum albumin (BSA-594; Thermo Fisher) was used. Before flow speed measurement, a standard curve of fluorescence intensity was created by measuring the average fluorescence intensity of a drop of different concentrations of BSA-594 on a coverslip. The solute concentration (50 μg/ml) used in this study was within the linear range of this curve (data not shown). The background fluorescence images were captured before the addition of fluorescent media and the time-lapse images were obtained every minute to monitor the perfusion of BSA-594. The maximum fluorescence images were captured after the imaging regions were uniformly perfused with BSA-594. Flow speed *v* was obtained using the following equation^69^.

1 $$v=\frac{L({I}_{2}-{I}_{1})}{({I}_{max}-{I}_{b})({t}_{2}-{t}_{1})},$$

where *﻿L* is the length of the measuring window,﻿ and *I*_*b*_ and *I*_*max*_ are background and maximum fluorescence intensity values, respectively. *I*_*1*_ and *I*_*2*_ are those taken at time *t*_*1*_ and *t*_*2*_, respectively.

Permeability of the agarose gel was measured using a dynamic mechanical analyzer (DMA, TA Instruments Q800) and post-analysis based on poroelastic theory as described previously^70^. Briefly, samples were prepared in a disk shape (12 mm-diameter and a 3 mm-thickness) and subjected to compression in the DMA based on a strain ramp from 0 to 10% at a rate of 10%/min. The reaction force exerted by the specimen on the compression clamp was recorded as the specimen was maintained at compression with constant strain (10%) for 120 min. Approximating the agarose gel as a poroelastic material, its force-relaxation behavior during the unconfined compression could be described as a function of its poroelastic properties, including permeability and elastic modulus, using a previously reported analytical model^71^. The permeability of agarose gel was then estimated by fitting the model simulations to the time-dependent force measurements using a non-linear optimization routine developed in MATLAB.

The shear stress (τ) applied on the cell by interstitial fluid flow was estimated by the following equation assuming spherical cells^72, 73^.

2 $$\tau =\frac{3}{\pi }(\frac{\mu Q}{A\sqrt{k}}),$$

where *μ* is the viscosity of the fluid, *Q* is the volumetric flow rate, *A* is the cross-sectional area of the flow chamber, and *k* is the Darcy permeability.

### Immunostaining and confocal microscopy

C28/I2 cells were mixed with AG, AG/Col, or AG-Col gels and cultured for 24 h before staining. The cell-gel constructs were fixed with 4% paraformldehyde. After rinsing, the cells were permeabilized with 0.5% Triton X-100 (Sigma) in PBS for 45 min at room temperature, and then incubated with blocking buffer (5% BSA, serum, 20% Polyvinylpyrrolidone (Amresco) in PBS combined into 1:1:1 ratio) overnight at 4 °C. The samples were incubated with primary antibodies against activated β1 integrin (clone HUTS-4; 1:500; Millipore) or total β1 integrin (clone P5D2; 1:100; Santa Cruz Biotechnology) overnight at 4 °C, and then with Alex Fluor 488 anti-mouse IgG (1:1000; Invitrogen) overnight at 4 °C. Finally, cell nuclei were labeled with 4′,6-Diamidino-2-phenylindole (DAPI; Sigma). An Olympus Fluoview FV1000 confocal microscope was used to visualize activated and total β1 integrins and cell nuclei. Images were acquired using a 60× objective lens (1.2 numerical aperture; Olympus). Cells were selected randomly. The fluorescence signal was quantified by measuring average intensity in individual cells using Fluoview Viewer software (Olympus).

### FRET microscopy

Images were obtained by using a Nikon Ti-E inverted microscope equipped with an electron-multiplying charge-coupled device (EMCCD) camera (Evolve 512; Photometrics), a filter wheel controller (Sutter Instruments), a Perfect Focus System (Nikon), and a heating and incubation system (Ibidi). The following filter sets (Semrock) were used: CFP excitation: 438/24 (center wavelength/bandwidth in nm); CFP emission (483/32); YFP (FRET) emission: 542/27. To minimize photobleaching, cells were illuminated with a 100 W Hg lamp through a neutral density 64 (~1.5% transmittance) filter. Images were acquired with a 40 × (0.75 numerical aperture) objective lens. FRET ratio images for SFK and FAK activities were generated with NIS-Elements software (Nikon) by computing an emission ratio of CFP/YFP for individual cells over time. Deconvolution (AutoQuant) was conducted to remove out-of-focus fluorescence signals and improve spatial resolution. Z-stack images with 0.3 μm-step size were obtained to generate orthogonal views. The representative FRET ratio images were produced using ImageJ (NIH) and scaled according to the color bar.

### Colocalization analysis

An Olympus Fluoview FV1000 confocal microscope was used to simultaneously visualize β1 integrin clustering and Lyn-FAK activity. Images were acquired using a 60× objective lens (1.2 numerical aperture; Olympus). Colocalization of β1 integrins and Lyn-FAK activity was calculated from randomly selected cells. Same cells were imaged before and after flow application. The images were first background-subtracted to reduce noise and then thresholded to highlight activities using ImageJ (NIH). The Pearson’s correlation coefficient was obtained using an ImageJ plugin JACoP^74^.

### Mouse cartilage explant culture and transfection

Experimental procedures were approved by the Indiana University Animal Care and Use Committee and were in compliance with the Guiding Principles in the Care and Use of Animals. C57BL/6 female mice (~12 weeks) were killed by inhalation of carbon dioxide. The femoral heads were isolated from the hip joint using a previously published procedure^75^. From the femoral surface, cartilage was surgically isolated. The explants were then transferred to 24-well plates and incubated overnight with DMEM supplemented with 10% serum and 1% penicillin/streptomycin. The *in vivo*-jet PEI (Polyplus) was used to transfect chondrocytes in cartilage explants with SFK and FAK biosensors following the manufacturer’s manual. Twelve hours after transfection, explants were mixed with AG-Col supplemented with DMEM to create 3D explant-agarose constructs. These constructs were incubated for ~12 hours before imaging experiments.

### Statistical analysis

Statistical data are presented as the mean ± standard error of the mean (SEM). One-way or two-way ANOVA were used to determine the statistical differences among multiple groups. Student’s t-test was used to compare two groups. Statistical analyses were conducted using Prism 5 software (GraphPad). A *p* value less than 0.05 was considered statistically significant.

### Data availability

The datasets generated during and/or analysed during the current study are available from the corresponding author on reasonable request.

## Electronic supplementary material

Supplementary Information
