# Supplementary material for: Subcellular domain-dependent molecular hierarchy of SFK and FAK in mechanotransduction and cytokine signaling
Source: Sci Rep. 2017 Aug 22;7:9033. doi: 10.1038/s41598-017-09495-5 (PMC5567257; doi:10.1038/s41598-017-09495-5)
Supplement: Supplementary file 1 — Supplementary Information [file 41598_2017_9495_MOESM1_ESM.pdf]

Supplementary Information:

Subcellular domain-dependent molecular hierarchy of SFK and FAK in mechanotransduction and cytokine signaling

Qiaoqiao Wan[1,2,6], ThucNhi TruongVo[1], Hannah E. Steele[1], Altug Ozcelikkale[3], Bumsoo Han[2,3], Yingxiao Wang[4], Junghwan Oh[5], Hiroki Yokota[1,2], Sungsoo Na[1,2,*]

[1]Department of Biomedical Engineering, Indiana University-Purdue University Indianapolis, Indianapolis, Indiana 46202, USA
[2]School of Biomedical Engineering, Purdue University, West Lafayette, Indiana 47907, USA
[3]School of Mechanical Engineering, Purdue University, West Lafayette, Indiana 47907, USA
[4]Department of Bioengineering, University of California San Diego, La Jolla, California 92093, USA
[5]Department of Biomedical Engineering, Pukyong National University, Busan 48513, Republic of Korea
[6]Present address: Department of Cell Biology, Harvard Medical School, Boston, Massachusetts 02115, USA

* Corresponding author: Sungsoo Na, PhD
Department of Biomedical Engineering
Indiana University-Purdue University Indianapolis
723 West Michigan Street, SL220G, Indianapolis, IN 46202, USA
Phone: 1-317-278-2384
Fax: 1-317-278-2455
E-mail: sungna@iupui.edu

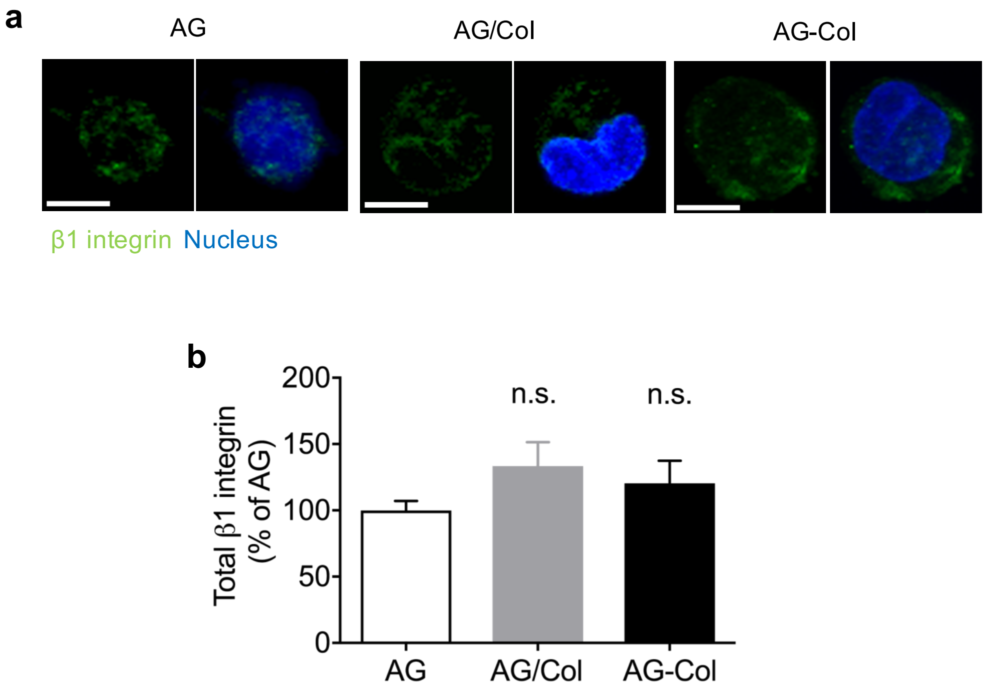

Figure S1. Total β1 integrin levels in cells grown in different types of agarose gels. (a) The representative immunostaining images of the total β1 integrins (green) and nuclei (blue) in AG, AG/Col, and AG-Col. Scale bars, 10 μm. (b) The bar graphs represent the GFP intensity of the total β1 integrins averaged over the whole cells in AG, AG/Col, and AG-Col. They were normalized against the averaged GFP intensity obtained in the AG. n = 8, 6, 8 in AG, AG/Col, AG-Col.

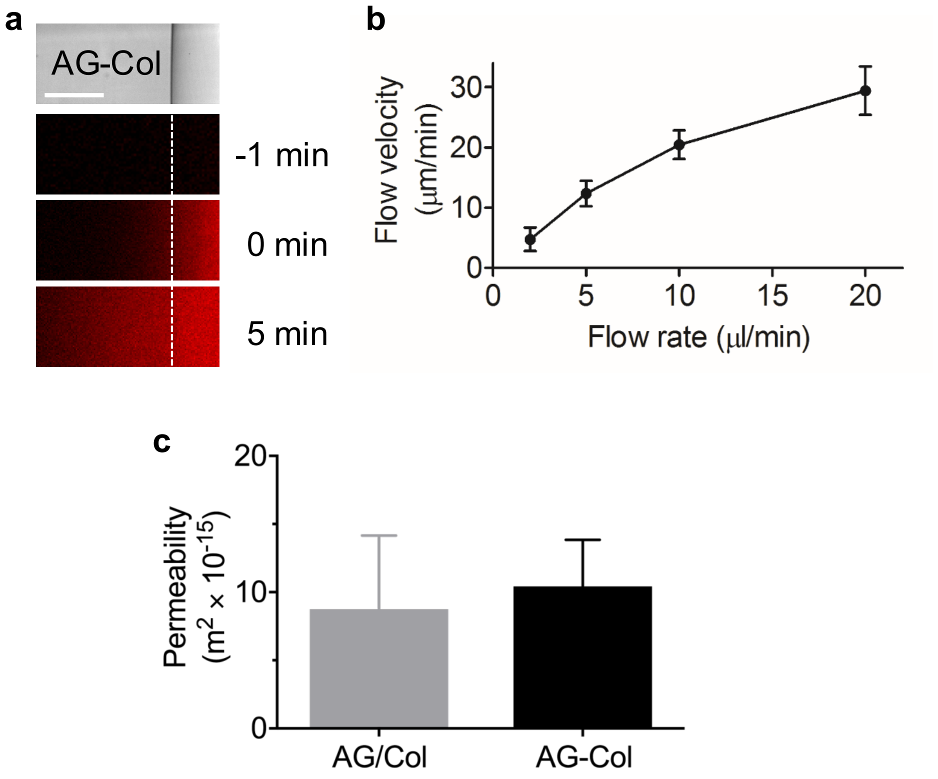

Figure S2. Flow velocity and permeability of collagen-conjugated agarose gels. (a) The representative images for the measurement of flow velocity (μm/min) through AG-Col under 20 μl/min fluid flow. Scale bar, 100 μm. (b) The flow velocity through AG-Col as under different flow rates. n = 12, 10, 10, 7 in 2, 5, 10, 20 μl/min. (c) The permeability values of the AG/Col and AG-Col. They were not significantly different. n = 4 in AB/Col and AG-Col.

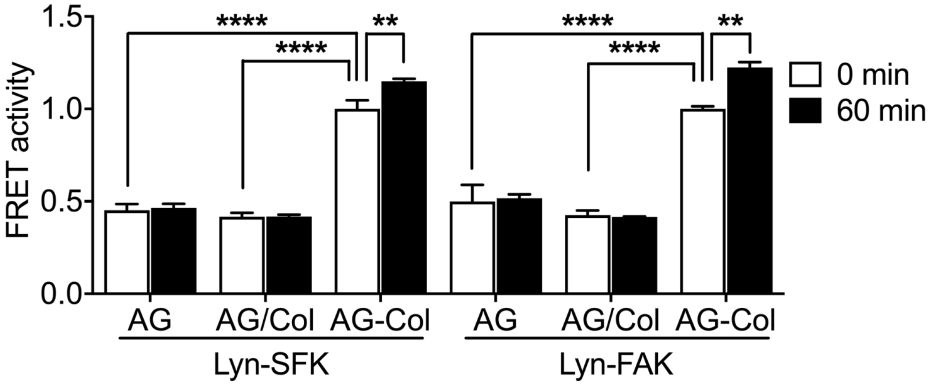

Figure S3. Lyn-SFK and Lyn-FAK activities before (0 min) and after (60 min) 20 µl/min fluid flow application in different types of gels. The activities were normalized to those at 0 min in AG-Col. Lyn-SFK, n = 7, 7, 10 in AG, AG/Col, AG-Col; Lyn-FAK, n = 7, 7, 21 in AG, AG/Col, AG-Col. ** $p < 0.01$. **** $p < 0.0001$.

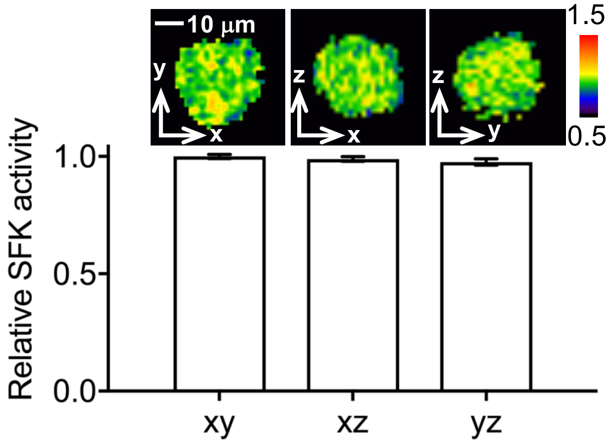

Figure S4. SFK activities from different orthogonal views have similar activation levels and patterns. Cells were transfected with the Lyn-SFK biosensor and z-stack images were obtained to generate FRET activity images in different projections. The bar graphs represent FRET ratios averaged over the whole cells and normalized to the averaged FRET ratios obtained from the x-y plane. *n* = 10, 10, 11 in xy, xz, yz.

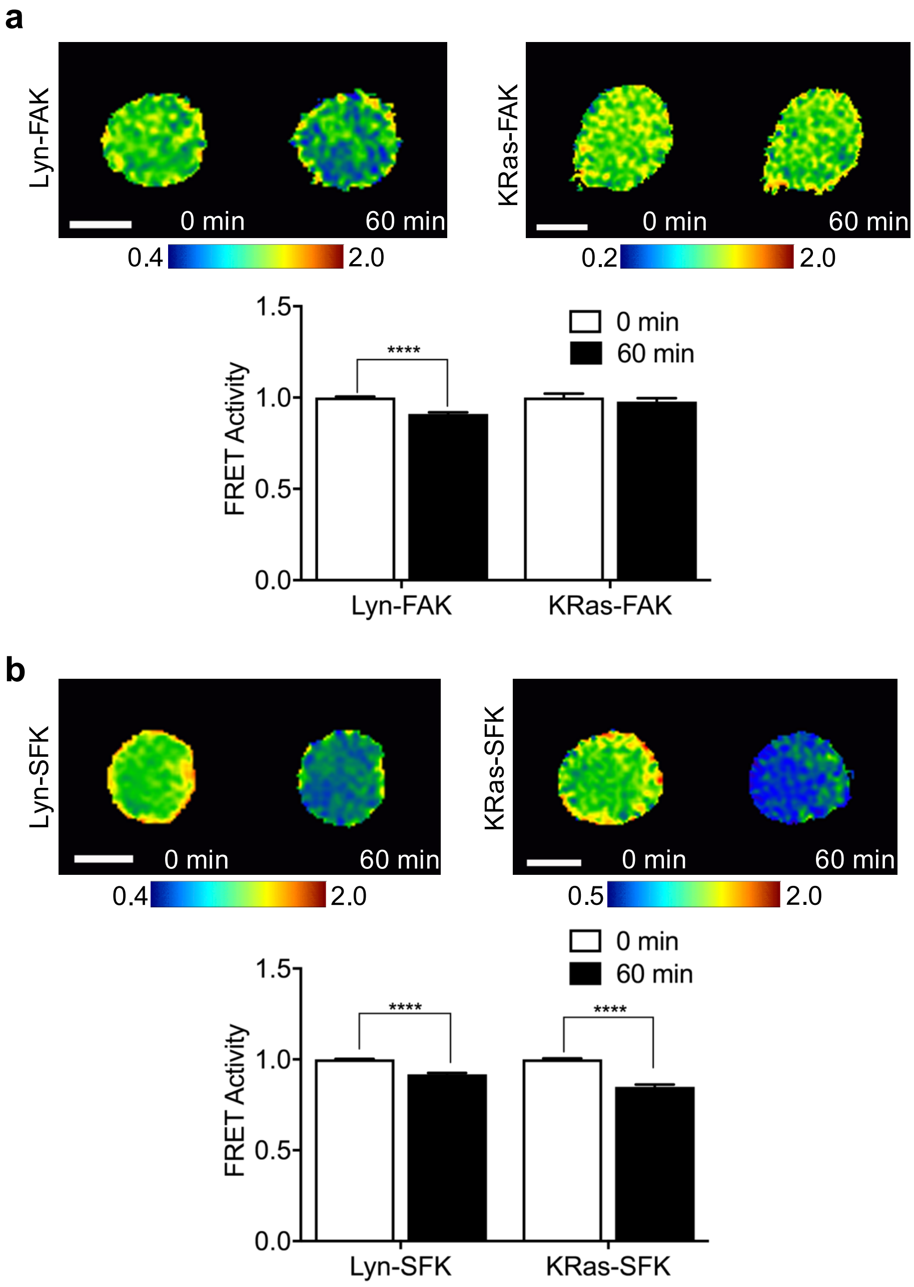

Figure S5. FAK and SFK activities at different subcellular domains in response to inhibiting drugs. (a) Lyn-FAK and KRas-FAK activities before (0 min) and after (60 min) treatment with 1 µM PF573228. Scale bars, 10 µm. The bar graphs represent FRET ratio images normalized to those at 0 min. Lyn-FAK, $n = 17$; KRas-FAK, $n = 9$. **** $p < 0.0001$. (b) Lyn-SFK and KRas-SFK activities before (0 min) and after (60 min) treatment with 10 µM PP2. Scale bars, 10 µm. The bar graphs represent FRET ratio images normalized to those at 0 min. Lyn-SFK, $n = 14$; KRas-SFK, $n = 23$. **** $p < 0.0001$.

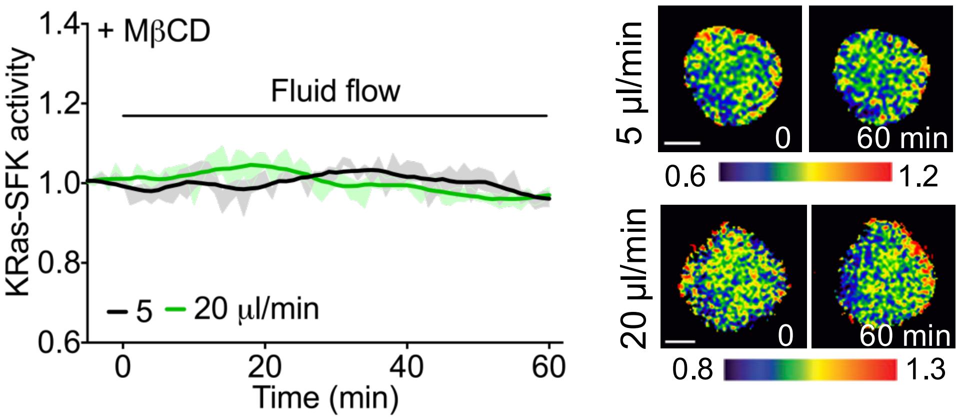

Figure S6. Lipid rafts are necessary for flow-induced KRas-SFK activity. Cells transfected with a KRas-SFK biosensor were pretreated with 10 mM MβCD for 1 h to disrupt lipid rafts in the plasma membrane. The time courses and representative images show that regulation of KRas-SFK by fluid flow was abolished by the treatment of MβCD. $n = 7$. Scale bars, 10 μm.

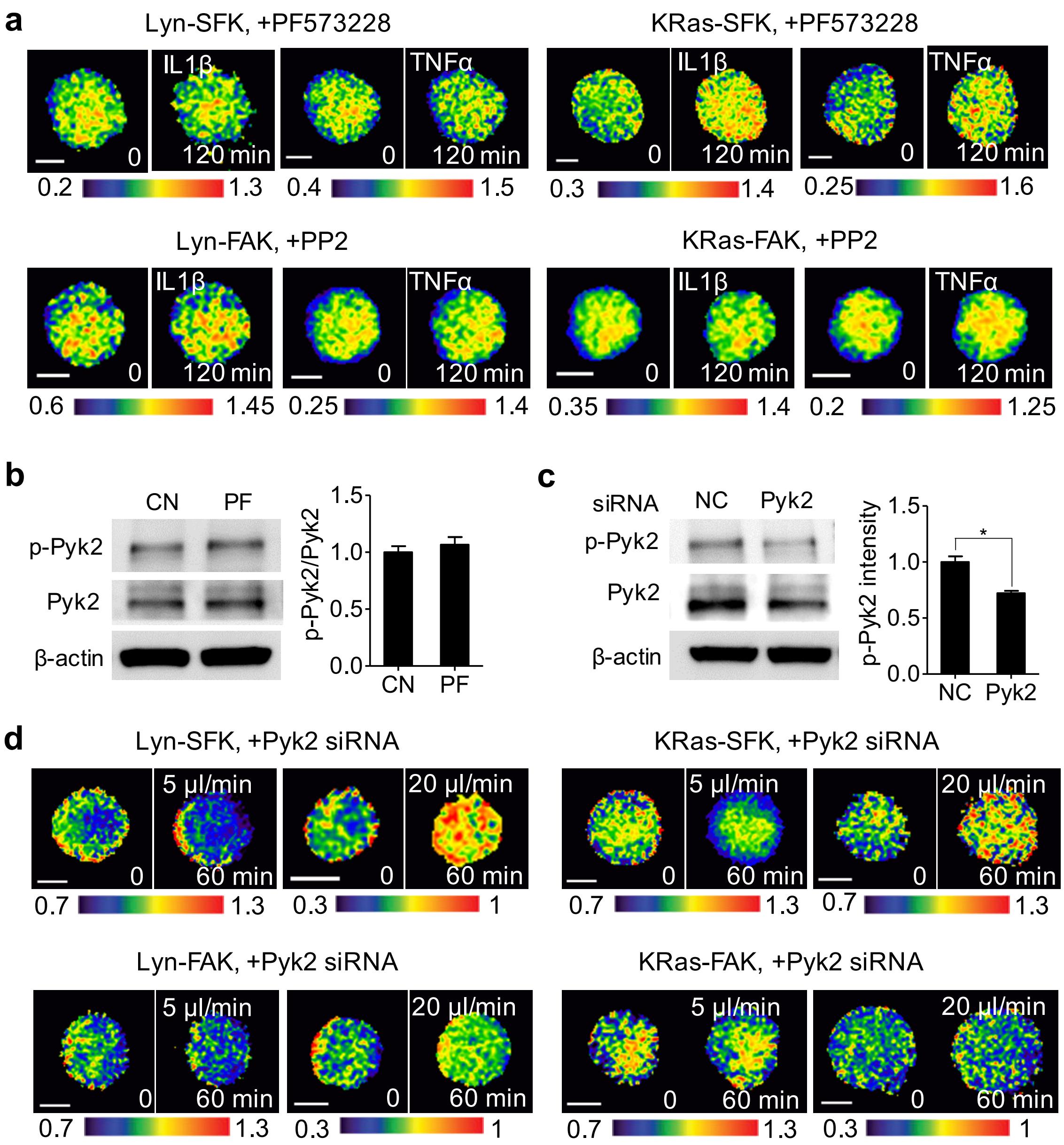

Figure S7. (a) The cytokine-induced activities of SFK and FAK at different subcellular domains in cells pretreated with FAK and SFK inhibitors. Cells transfected with either a Lyn-SFK or KRas-SFK biosensor were pretreated with PF573228. Cells transfected with either a Lyn-FAK or KRas-FAK biosensor were pretreated with PP2. (b) Effects of PF573228 (PF, 1 μM) on the phosphorylation and expression levels of Pyk2. CN indicates control. $n = 4$ per group. (c) Effects of Pyk2 silencing on the phosphorylation and expression levels of Pyk2. NC indicates the non-specific control siRNA. $n = 4$ per group. * $p < 0.05$. (d) The flow-driven activities of SFK and FAK at different subcellular domains in cells pretreated with Pyk2 siRNA. Cells were co-transfected with Pyk2 siRNA and one of the FRET biosensors, and then subjected to 5 or 20 μl/min fluid flow for 1 hour. Scale bars, 10 μm.

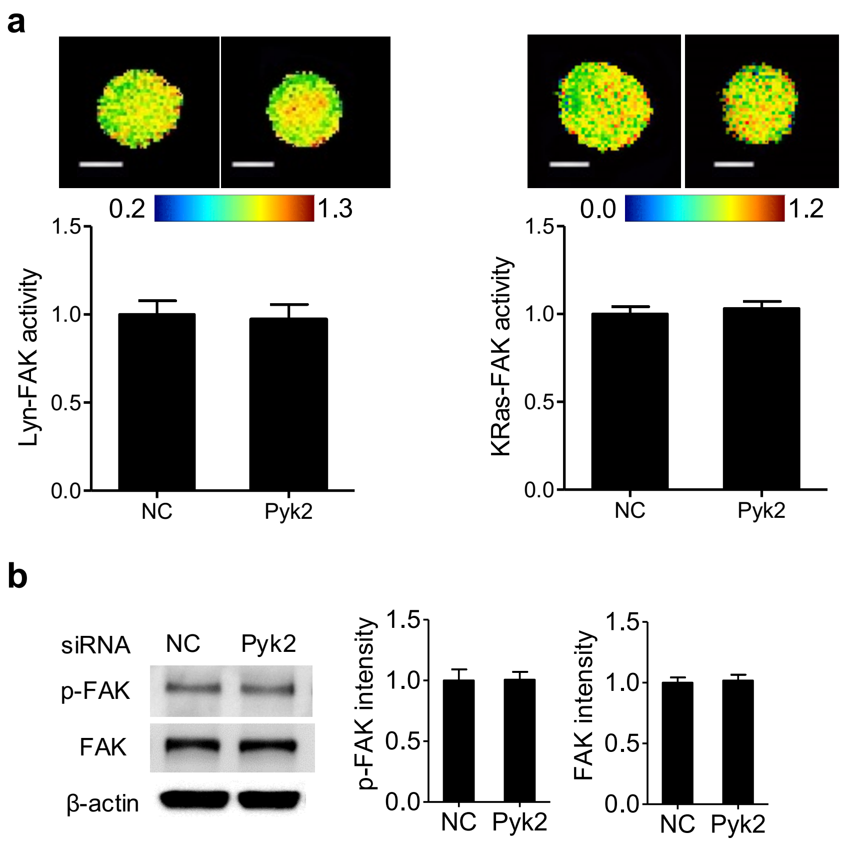

Figure S8. Partial inhibition of Pyk2 does not affect FAK activity. (a) The basal activity of Lyn-FAK and KRas-FAK in response to Pyk2 knock down. Cells were co-transfected with the FAK biosensor (Lyn-FAK or KRas-FAK) and either non-specific control (NC) or Pyk2 siRNA. Both Lyn-FAK and KRas-FAK were not altered by Pyk2 siRNA. For Lyn-FAK, n = 21, 17 in NC, Pyk2. For KRas-FAK, n = 16, 17 in NC, Pyk2. Scale bars, 10 µm. (b) Effects of Pyk2 silencing on the phosphorylation and expression levels of FAK. n = 4 per group.
